# Metabolic crosstalk in the ageing brain: astrocyte-neuron coupling as a target for homeostatic restoration and therapy

**DOI:** 10.1186/s40035-026-00562-4

**Published:** 2026-06-22

**Authors:** Lihui Qian, Yanting Deng, Meiying Song, Zhouyuan Zhang, Jiawei Cheng, Boran Zhu, Minghua Wu, Haosu Zhang, Yue Hu

**Affiliations:** 1 Department of Neurosurgery, Anhui Digital Brain Engineering Research Center& The First Affiliated Hospital of Wannan Medical College, Yijishan Hospital of Wannan Medical College, Wuhu, 241001 Anhui People’s Republic of China; 2 Nanjing University of Chinese Medicine, Nanjing, 210023 China; 3 Department of Cardiology, The Second Affiliated Hospital, School of Medicine, Zhejiang University, Hangzhou, 310009 China; 4 State Key Laboratory of Transvascular Implantation Devices, Hangzhou, China; 5 Heart Regeneration and Repair Key Laboratory of Zhejiang Province, Hangzhou, China; 6 Department of Neurology, Affiliated Hospital of Nanjing University of Chinese Medicine, Jiangsu Province Hospital of Chinese Medicine, Nanjing, 210029 China

**Keywords:** Regional, Astrocyte-neuron, Metabolic coupling, Ageing, Targeted therapies

## Abstract

Astrocytes, the most abundant glial cells in the central nervous system, play increasingly recognized roles in metabolic regulation beyond their classical supportive functions. This review summarizes current understanding of astrocyte morphological and molecular heterogeneity, with emphasis on regional metabolic properties across different brain areas. We discuss the primary forms and functional significance of astrocyte-neuron metabolic coupling, focusing on glucose, lipid, and glutamate metabolism, lactate shuttling, as well as the mitochondrial reactive oxygen species signaling. Furthermore, we integrate emerging evidence from animal and preclinical studies linking astrocyte-neuron metabolic coupling to ageing-related diseases. Collectively, these findings highlight how astrocyte–neuron interactions sustain physiological homeostasis and contribute to pathological manifestations, underscoring their significance in both health and ageing-related diseases.

**Graphical abstract:**

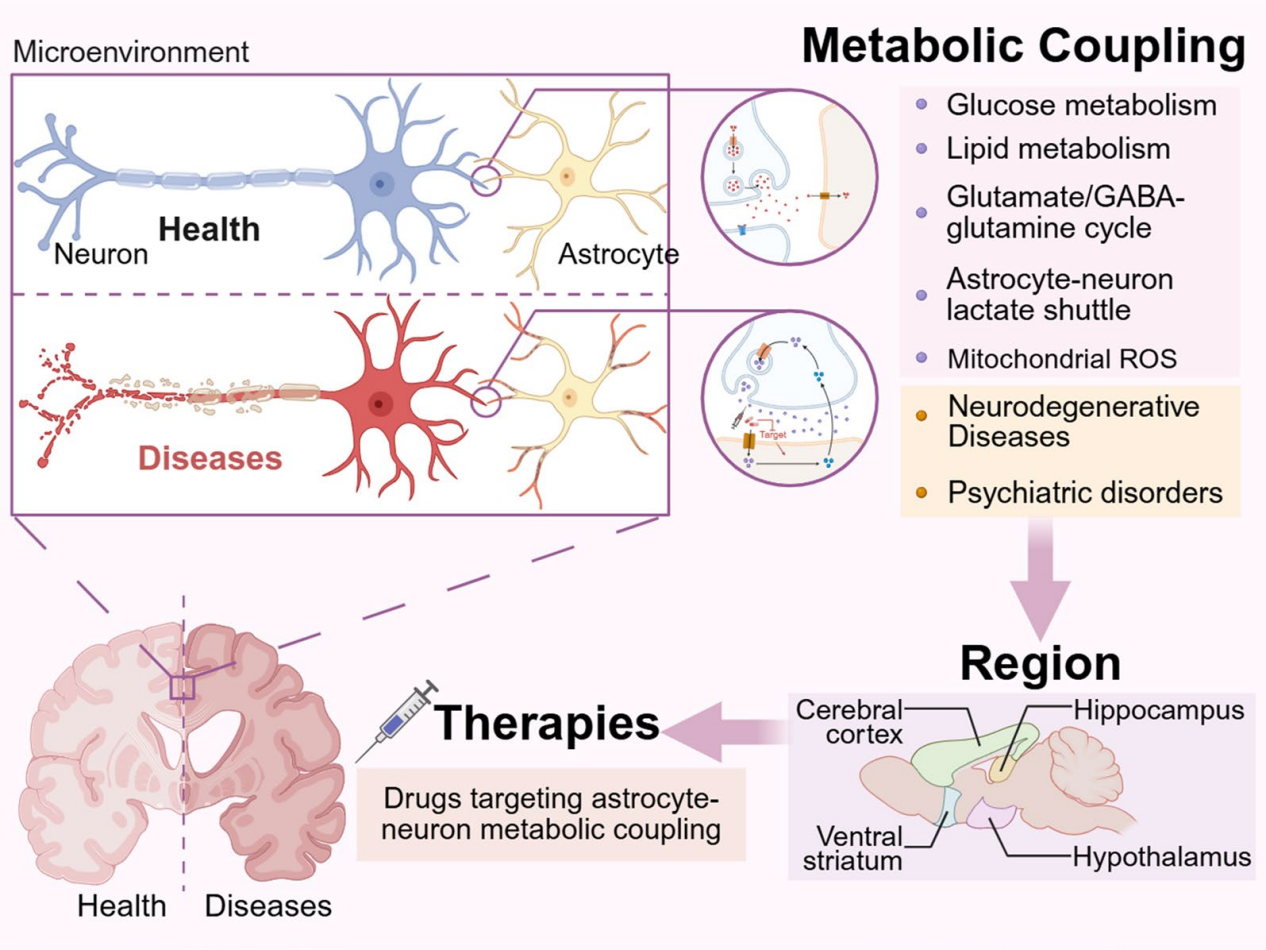

## Introduction

Astrocyte-neuron metabolic coupling plays a crucial role in maintaining brain function and is emerging as a novel therapeutic direction for central nervous system (CNS) disorders [1, 2]. Astrocytes provide neurons with essential metabolic substrates, enabling efficient resource allocation for high-level activity during information processing. Astrocytes also participate in neurotransmitter recycling, synaptic cleft clearance, and maintenance of a healthy neuronal microenvironment while protecting neurons from potential neurotoxic effects [3, 4]. Disruptions in this coupling are closely associated with various CNS disorders.

The role of astrocyte-neuron metabolic coupling in maintaining cerebral energy homeostasis has been extensively studied. However, the brain regional heterogeneity in cellular density, synaptic activity, and energy demands suggests the likely evolution of distinct coupling strategies [5]. For instance, the glutamate provided by astrocytes can be utilized by hippocampal neurons to consolidate memories, while the energy derived from the glucose metabolism of astrocytes can sustain the functions of the hypothalamus [6, 7]. These regional metabolic coupling patterns and their age-dependent vulnerabilities become particularly evident in neurological disorders, as seen in the characteristic hippocampal hypometabolism in Alzheimer’s disease (AD) and the striatum-specific metabolic dysfunction of astrocytes in Huntington’s disease (HD) [8].

Building upon these insights, we review astrocytic morphology and molecular signatures, and analyze how these specialized coupling mechanisms, which are essential for whole-brain metabolic homeostasis, become differentially vulnerable to the ageing process. Specifically, we examine how age-associated dysregulation of these couplings creates distinct, region-specific metabolic vulnerabilities that predispose to various CNS disorders. Understanding how the brain’s metabolic architecture evolves with age provides scientific foundations for developing interventions that target the ageing processes to restore metabolic resilience.

## Spatial heterogeneity of astrocytes

Astrocytes demonstrate remarkable morphological and molecular heterogeneity across CNS regions, developing distinct structural and functional specializations shaped by their microenvironment. This diversity, spanning from regional branching patterns to molecular subpopulations with unique metabolic profiles, underpins the critical roles of astrocytes in neural circuit regulation and brain homeostasis.

### Morphological diversity of astrocytes

Astrocytes originate from neural progenitor cells in the ventricular zone and migrate to distinct brain regions, where they differentiate into diverse morphological subtypes [9]. Their intricate and densely branched architecture enables long-range contacts and signaling with various cell types, thereby facilitating functional roles including synaptic modulation, neuronal circuit responsiveness, and the establishment of CNS homeostasis [10].

Astrocytes exhibit morphological heterogeneity across the CNS, with two principal subtypes: protoplasmic astrocytes, which are characterized by short, highly branched processes predominantly localized in gray matter, and fibrous astrocytes, which possess elongated, sparsely branched processes primarily residing in white matter [8]. Furthermore, interlaminar astrocytes, which are specifically localized to Layer I of the cerebral cortex, exhibit distinct morphological features, including small cell bodies and long, fasciculated processes [11]. Astrocytes consist of a soma and radially projecting processes, exhibiting a dense, “spongiform” morphology. Research shows that astrocytes can be arranged in a flat 2D pattern within a region, with an astrocyte territory overlap rate of less than 5% at their edges, and they may be able to interlock the entire CNS in 3D [12].

Studies of the mechanisms of astrocyte morphogenesis have advanced recent years. Early studies identified that neuronally secreted factors, including fibroblast growth factor, brain-derived neurotrophic factor, and glutamate, play pivotal roles in shaping astrocyte morphogenesis [13, 14]. Recent studies have focused on the regulation of astrocyte morphogenesis by a variety of cell adhesion molecules through a contact-mediated mechanism, revealing that these molecules affect astrocyte infiltration, branching complexity, and synaptic function with spatial–temporal and layer-specific specificity. In addition, the astrocyte-neuron and astrocyte-astrocyte interactions are coordinated by molecules such as cadherins and gap junctions, which jointly regulate the morphological maturation and synaptic plasticity of astrocytes [10].

Through single-cell RNA sequencing, transcriptomics, and other research techniques, researchers have determined that astrocytes can be divided into multiple subsets. By quantifying key morphological metrics such as astrocyte area size, branching structure, and fractal dimension, researchers found that astrocytes in the motor cortex exhibit more rounded regional shapes and are larger than those in the striatum, whereas cerebellar astrocytes exhibit elongated morphology. These results suggest regional heterogeneity of astrocyte morphology and its potential functional differences [15]. The morphological diversity of astrocytes is essential for their functional diversity. Gene set enrichment analysis revealed that protoplasmic astrocytes are enriched for genes related to steroid metabolism, cholesterol biosynthesis, and neurotransmitter release [16]. In addition, astrocytes interact with synapses through gap junctions to facilitate the transport and communication of neurotransmitters, lactate, and other metabolites. By responding to environmental changes, astrocytes play a crucial role in regulating the whole-body energy metabolism [2, 17].

### Molecular heterogeneity of astrocytes

Astrocytes exhibit profound molecular heterogeneity in different brain regions, which fundamentally determines their functional diversity and metabolic interactions with neurons. Cortical astrocytes show an enrichment of genes related to synaptogenesis and glutamate processing compared to astrocytes in other regions [18], whereas hypothalamic astrocytes are associated with expression of genes related to metabolic homeostasis, such as cholesterol metabolism and neurogenesis [19]. Transcriptomic analysis revealed that astrocytes from the cortex, hippocampus, olfactory bulb, and cerebellum all show unique patterns of gene expression [18, 20]. In addition to regional differences, astrocytes exhibit significant subregional heterogeneity, even within a single brain structure. For example, in hippocampal subregions cornu ammonis (CA) and dentate gyrus (DG), the molecular profiles of astrocytes may reflect their local functional requirements [21]. Single-cell transcriptomic analysis has further divided CNS astrocytes into seven distinct subsets (AST1–AST7), each showing unique regional distribution patterns and metabolic pathway enrichment [15]. Notably, AST2 and AST4–AST6 exhibit upregulated glucose metabolism, AST2 exhibit enhanced cholesterol homeostasis, and neurotransmitter homeostasis pathways are differentially enriched between AST1–AST6. This highlights how astrocyte subclass-specific metabolic specialization contributes to the maintenance of local brain homeostasis [15].

The molecular structure of astrocytes undergoes significant remodeling during ageing. Mononuclear transcriptome analysis of the ageing mouse brain revealed cell type-specific changes that differed significantly across regions [22]. In the ageing hypothalamus, astrocytes exhibit distinct transcriptional profiles characterized by reduced enrichment of cholesterol homeostasis genes [19]. In addition, Astro-TE_5, a telencephalic astrocyte supertype identified by single-cell analysis, showed significantly decreased expression of *Csmd1* (a gene associated with cognitive decline and neurodegenerative diseases [23]) with age. The abundance of specific astrocyte subtypes also varies with age. The major telencephalic astrocyte subtype Astro-TE_3 remained relatively stable, while the major non-telencephalic astrocyte subtype Astro-TE_2 showed significant age-related reductions in the midbrain and hindbrain [22]. Reactive astrocyte features appear preferentially in certain regions, suggesting that ageing does not uniformly affect all astrocytes, but rather targets specific molecularly defined subsets. This regional heterogeneous ageing response may underlie the differences in the responses of different brain regions to age-related neurodegenerative diseases.

An evident functional consequence of astrocyte molecular heterogeneity and its age-related changes is the alterations in intercellular communication. Young astrocyte-neuron pairs demonstrate abundant signaling through multiple pathways, including TGFB2-TGFBR3 and FGF9-mediated signaling. These pathways involved in repair, learning, memory, and neurogenesis are progressively lost with age [19]. Similarly, aged astrocyte-neuron pairs show reduced SEMA3A-NRP1 signaling, a key pathway in synaptogenesis [19, 24]. The loss of these astroglia-derived signals may have contributed to the altered synaptic homeostasis in the ageing brain.

The molecular heterogeneity of astrocytes underlies their morphological diversity, manifested as regional expression of genes and proteins that shape their functionally relevant metabolic pathways, providing new insights into their role in brain health and disease.

### Regional astrocyte-neuron molecular heterogeneity

Distinct astrocyte subtypes and subpopulations exhibit diverse functional states. The hippocampus is an example of subregional astrocyte specialization [25]. The hippocampal subregions CA1, CA2, CA3, and DG are closely associated with adult neurogenesis and cognitive functions. CA1 astrocytes serve as regulators of the primary output pathway and play a crucial role in hippocampus-dependent memory storage through specialized interactions with synapses [26, 27]. In different regions of the hippocampus, a specific subpopulation of astrocytes selectively mediates glutamatergic glial transmission [7]. Absence of vesicular glutamate transporter (VGLUT) 1 in these glutamatergic astrocytes leads to deficits in long-term potentiation and memory within the cortico-hippocampal circuit [7]. In the ventral hippocampus, astrocytes maintain neurotransmission and neuroprotection through glutamate transporters (GLTs), such as GLT-1. These transporters recapture glutamate from the synaptic cleft into astrocytes, thereby regulating glutamate levels and preventing excessive excitation and neurotoxicity [28].

The amygdala and striatum show state-dependent astrocytic transformations. In the basolateral amygdala (BLA), astrocytes release glutamate and *D*-Ser through the Cx43 gap junction to activate postsynaptic N-methyl-D-aspartic acid receptors (NMDARs) [29], thereby facilitating fear memory consolidation. This represents a physiological state of enhanced glial transmission during emotional learning. Moreover, circadian clock molecules in nucleus accumbens (NAc) astrocytes participate in the regulation of reward-related behaviors [30], suggesting a time-dependent state transition. Two-photon laser scanning microscopy has been employed to investigate the reduction in Ca^2+^-dependent signaling in striatal astrocytes. Studies have found that blocking the astrocytic GABA transporter 3 alters striatal microcircuitry, leading to obsessive-compulsive disorder-like behaviors in mice [31]. Additionally, astrocytic VGLUT2 deficiency affects the nigrostriatal circuit function and dopamine levels *in* *vivo* [32]. This transition from homeostatic support to a pathological state in the striatum demonstrates that transporter dysfunction in specific astrocyte populations can drive behavioral abnormalities.

Single-cell RNA sequencing has revealed remarkable molecular heterogeneity among cortical astrocytes [18]. The morphological development of cortical astrocytes depends on direct contact with neuronal processes and unfolds in parallel with synaptic circuit maturation. Neuroligin-2 deficiency in astrocytes significantly impairs the formation and function of excitatory synapses in the cortex, while inhibitory synaptic functions are enhanced [33]. This suggests that molecularly distinct astrocyte populations may differentially regulate excitatory and inhibitory synaptic development, thereby shaping the cortical circuit balance.

## Main forms and functions of astrocyte-neuron metabolic coupling

**Fig. 1:**
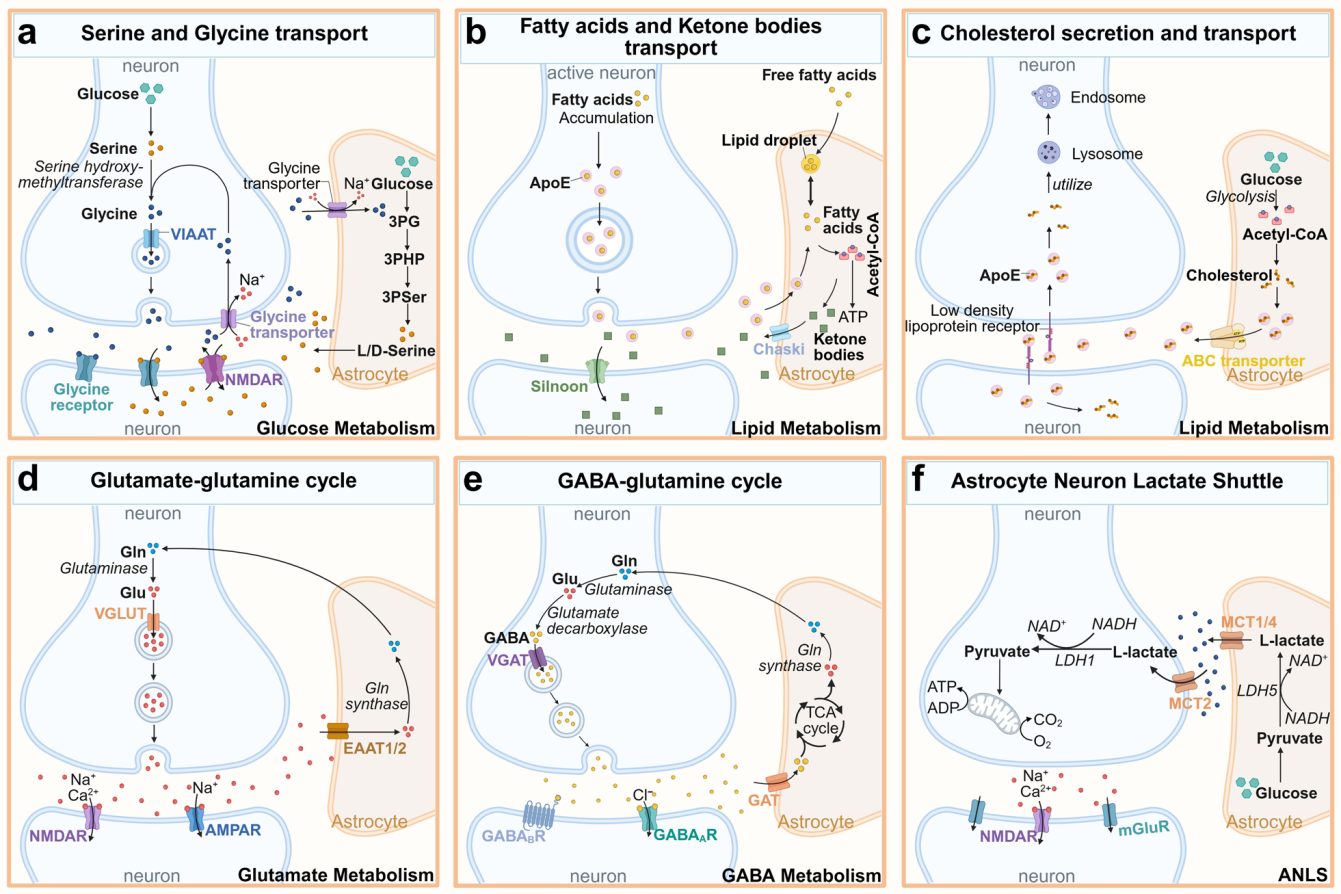
Forms of astrocyte-neuron metabolic coupling. **a** Synthesis and transport of serine and glycine in glucose metabolism. **b** Transport of fatty acids and ketone bodies in lipid metabolism. **c** Cholesterol secretion and transport in lipid metabolism. **d** The metabolic glutamate-glutamine cycle between astrocytes and neurons. **e** The metabolic cycle of GABA and glutamine between astrocytes and neurons. **f** The metabolic lactate shuttle between astrocytes and neurons. 3PG: 3-Phosphoglyceric acid; 3PHP: 3-Phosphohydroxypyruvate; 3PSer: 3-Phosphoserine; ANLS: Astrocyte-neuron lactate shuttle; EAAT: Excitatory amino acid transporter; GAT: GABA transporter; GABA: γ-Aminobutyric acid; Glu: Glutamate, Gln: Glutamine; GLUT: Glucose transporter; MCT: Monocarboxylate transporter; mGLUR: Metabotropic glutamate receptors; NMDAR: N-methyl-D-aspartate receptor; VGAT: Vesicular GABA transporter; VGLUT: Vesicular glutamate transporter; VITTA: Vesicular inhibitory amino acid transporter

In this section, we discuss five major modes of astrocyte-neuron metabolic coupling. These tightly regulated interactions rely on a marked cell-type specialization in glycolysis and redox control: astrocytes are endowed with robust glycolytic competence (including nitric oxide-AMPK-PFKFB3 [6-phosphofructo-2-kinase/ fructose-2, 6-bisphosphatase-3]-dependent control) [34], whereas neurons actively suppress PFKFB3 through anaphase-promoting complex/cyclosome (APC/C)-Cdh1-mediated proteasomal degradation to maintain a physiologically hypoglycolytic state that preserves the pentose phosphate pathway (PPP)-dependent antioxidant defense [35]. In parallel, cell-specific mitochondrial organization (including complex I assembly into supercomplexes) shapes the differential mitochondrial reactive oxygen species (ROS) production and signaling in neurons versus astrocytes [36]. Importantly, this compartmentalization is functionally required *in* *vivo*, since enforcing neuronal glycolysis disrupts mitochondrial fitness and impairs cognition [37] (Fig. 1).

### Glucose metabolism

The glucose-related metabolic interaction between astrocytes and neurons regulates the function and behavior of neural circuits [1, 38, 39].

#### Serine (Ser) and glycine (Gly) shuttle

*L*-Ser/*D*-Ser plays an important role in astrocyte-neuron interaction [40], learning and memory [41], and synaptic plasticity [42, 43]. Ser biosynthesis originates from glycolytic intermediates. In the glycolytic metabolic pathway, the intermediate 3-phosphoglycerate is dehydrogenated to 3-phosphohydroxypyruvate under the action of phosphoglycerate dehydrogenase. The 3-phosphohydroxypyruvate is sequentially converted to 3-phosphoserine, eventually yielding Ser [44]. Ser produced by astrocytes activates glutamate receptors NMDARs on neurons to participate in the regulation of brain function [1]. When the cellular Ser level reaches a sufficient threshold, pyruvate kinase M2 (PKM2) is activated to facilitate glycolysis. Conversely, in the event of Ser deficiency, pyruvate kinase activity mediated by PKM2 diminishes, leading to the accumulation of 3-phosphoglycerate molecules, which subsequently enter the Ser synthesis pathway to support cell proliferation [44]. Furthermore, neuronal Ser can be metabolized into Gly, which is involved in subsequent metabolic pathways. The neuron-derived Gly activates Gly receptors expressed on astrocytes, playing a functional role in the metabolic coupling between astrocytes and neurons [45] (Fig. 1a).

#### Glucose transport

The cellular uptake of glucose by astrocytes is mediated by the glucose transporter GLUT1, and the sodium–potassium pump facilitates the entry and utilization of glucose. Subsequently, the generated energy substrates and lactate are supplied to neurons to meet their metabolic demands [46]. Neurons can take up extracellular glucose through GLUT3 for energy metabolism [1]. Furthermore, under hypoxic environments, astrocytes contribute to cerebral vasodilation and enhanced blood perfusion, which augments the uptake of glucose by neurons via GLUT3 [47]. Interestingly, recent studies have shown that deletion of GLUT1 specifically in adult astrocytes does not impair motor and memory functions. In the absence of GLUT1, astrocytes adapt their glucose uptake machinery to markedly increase glucose uptake and consumption, demonstrating that these cells possess robust metabolic resilience to maintain glucose supply and neuronal support even when a major transporter is absent [48]. This finding indicates the existence of an alternative GLUT in astrocytes capable of compensating for glucose uptake and metabolism, a mechanism that warrants further investigation (Fig. 1a).

#### Indirect transport of metabolites

A seminal paper highlighted the role of astrocytes in facilitating the transport of glucose and its metabolites to distal neurons through gap junction subunit proteins connexin (Cx) 43 and Cx30. This activity-dependent intercellular transport process is regulated by AMPA receptor-mediated glutamatergic synaptic activity [49]. Furthermore, it has been revealed that the high expression of Cx43 and Cx30 on hypothalamic astrocytes contributes to heightened glucose sensitivity and facilitates increased insulin secretion [50]. Interestingly, the regulation of Cx43 and Cx30 expression on astrocytes is intricately controlled by neuronal signaling pathways [51].

In summary, astrocytes metabolize glucose mainly into lactate through aerobic glycolysis [34, 35]. This not only provides energy substrates and neuroregulatory molecules for neurons, but also forms complementary metabolic coupling with neurons through multiple mechanisms, such as metabolite delivery to jointly support advanced brain functions by maintaining brain energy homeostasis and synaptic plasticity.

### Lipid coupling

Lipids encompass triglycerides and lipoids. The triglycerides are hydrolyzed into glycerol and fatty acids (FAs) during fat mobilization, for oxidation and utilization by other tissue cells. Lipoids, meanwhile, include cholesterol and phospholipids, among other lipid species. Furthermore, lipid metabolism is closely intertwined with glucose metabolism. Glucose can be metabolized to produce acetyl-CoA, a key precursor that enters the de novo synthesis pathway, leading to the production of FAs and cholesterol. This metabolic interplay underscores the complexity of energy management within the brain and the potential implications for neurological health [52, 53].

#### FAs

The coordination of FAs metabolism represents a pivotal aspect of the functional interplay between neurons and astrocytes [54, 55]. FAs are key components of fat, phospholipids, and cholesterol esters, and are found within lipid droplets (LDs) in cells [54]. Under the condition of sufficient oxygen supply, FAs enter the tricarboxylic acid (TCA) cycle to generate large amounts of ATP.

Notably, neurons typically do not generate LDs and exhibit low levels of energy consumption from FA oxidation in their mitochondria [56, 57]. Under highly active states, neurons accumulate toxic FAs, and transfer FAs to astrocytes through secretion of apolipoprotein E (ApoE) lipid particles. The astrocytes form a neuroprotective metabolic coupling with neurons by β-oxidation of these FAs in mitochondria, effectively alleviating the neuronal activity-dependent lipotoxic stress. Studies have shown that when neurons are highly active, their mitochondria produce high levels of ROS, inducing lipid peroxidation and accumulation of FAs in neurons through autophagy [58, 59]. In neurons, toxic FAs are not stored in LDs but are excreted from neuronal cells through the endoplasmic reticulum and Golgi apparatus to form ApoE-positive lipid particles. Interestingly, ApoE does not wander around the cells at will but is absorbed by neighboring cells [54]. To this end, a study developed Red-C12 transfer experiments to confirm that the labeled FAs in neurons can be transferred to glial fibrillary acidic protein-positive astrocytes and accumulate in the LDs of astrocytes in the absence of cell contact [54]. Astrocytes consume FAs through mitochondrial β-oxidation and eventually undergo oxidative phosphorylation (OXPHOS) to achieve detoxification (Fig. 1b) [53].

#### Ketone bodies (KBs)

Astrocytes convert FAs into KBs [53, 60, 61] and supply neurons with energy for oxidation through Chaski/Silnoon transporters [62, 63]. This serves as an alternative energy pathway in the absence of glucose to maintain brain energy homeostasis and achieve the metabolic cooperation of “astrocytic ketogenesis and neuronal oxidation”. This process is regulated at two layers. Under high glucose conditions, malonyl-CoA, a key metabolic derivative of acetyl-CoA, accumulates and competitively inhibits KB synthesis by suppressing the activity of carnitine palmitoyltransferase I at the mitochondrial outer membrane [60]. In addition, KB production and transport are mediated by AMP-activated protein kinase and regulated by cyclic AMP. During hypoxia and glucose deprivation, cyclic AMP activates protein kinase A, which phosphorylates and inactivates acetyl-CoA carboxylase, thereby promoting astrocyte ketogenesis and reducing malonyl-CoA levels [60] (Fig. 1b).

#### Cholesterol

Studies utilizing Gene Set Enrichment Analysis have identified an enrichment of genes associated with cholesterol biosynthesis and metabolism in cortical astrocytes, particularly in protoplasmic astrocytes, highlighting their close interaction with neurons [16]. Astrocytes synthesize the major cholesterol in the brain through the acetyl-CoA pathway and secrete apolipoprotein ApoE to mediate its transport to neurons. The cholesterol synthesized in astrocytes is packaged onto ApoE, which then utilizes ATP-binding cassette transporters to facilitate its movement [64, 65]. This cholesterol, once secreted as lipidated ApoE particles, is internalized by neurons through a receptor-mediated endocytosis process that engages the low-density lipoprotein receptor and low-density lipoprotein receptor-related protein 1 [66]. The exogenous cholesterol is vital for neuronal function, as it is directed to endosomes and lysosomes within neurons [66, 67]. Strikingly, maintenance of neuronal cholesterol within a precise range is essential. Neuronal cholesterol deficiency potentially impairs memory formation and its excess disrupts synaptic plasticity, ultimately leading to neuronal apoptosis [68–70] (Fig. 1c).

In summary, astrocytes and neurons are functionally connected through a complex lipid metabolism network. Astrocytes not only remove toxic FAs produced by neurons through β-oxidation, but also convert FAs into KBs to supply neurons with energy when glucose is deficient [55]. Astrocytes also dominate the synthesis of cholesterol in the brain and deliver cholesterol to neurons via the ApoE pathway.

### Glutamate/GABA–glutamine cycle

Glutamate is synthesized directly from the TCA cycle intermediate α-ketoglutaric acid. This conversion is primarily catalyzed by glutamate dehydrogenase and aspartate aminotransferase [71]. The process of glutamatergic excitatory synaptic transmission is facilitated by various transporters, including vesicular transporters and GLTs that ensure the efficient release and uptake of glutamate. VGLUT1–3 are instrumental in loading glutamate into synaptic vesicles. Once glutamate is loaded into these vesicles, they are released from presynaptic terminals [72]. On the plasma membrane, a group of transporters known as the solute carrier 1 family is responsible for the uptake of glutamate from the extracellular fluid into the cytoplasm. Among these transporters, excitatory amino acid transporter 1 and 2 (EAAT1 and EAAT2) are predominantly expressed in astrocytes, playing a crucial role in maintaining glutamate homeostasis [73, 74]. Once glutamate is taken up by astrocytes via these transporters, it is metabolized to glutamine by the enzyme glutamine synthetase (GS), thereby completing the cycle. Notably, recent findings demonstrate that astrocytes dynamically modulate their responsiveness to glutamate through G protein-coupled adrenergic signaling [75] (Fig. 1d).

In GABAergic neurons, glutamate is transformed into GABA by the rate-controlling enzyme glutamate decarboxylase [76]. Following its release into the synaptic cleft via GABA transporters, GABA activates GABA_A_ receptors localized on astrocyte membranes, enabling its uptake into these glial cells [77]. In contrast to the glutamate metabolism, the process of GABA metabolism relies on only two enzymes: GABA-transaminase and succinic semialdehyde dehydrogenase (SSADH), which catalyze the irreversible conversion of GABA into the TCA cycle intermediate succinate for further oxidation [71]. A study has highlighted the critical role of GABA metabolic homeostasis in synaptic activity by demonstrating that a deficiency in SSADH leads to aberrant expression of proteins associated with amino acid homeostasis, mitochondria, and glial function [78] (Fig. 1e).

During the process of glutamate/GABA–glutamine cycle, as discussed above, glutamate and GABA are both taken up by astrocytes after their release into the synaptic cleft, thus propagating neural signals [79]. Subsequently, glutamine synthesis occurs specifically within these homeostatic glial cells. This process is catalyzed by GS. Notably, *L*-methionine sulfoximine, which inhibits the activity of GS, reduces neuronal synthesis of glutamate and GABA, leading to disrupted neuronal signaling [80]. This underscores the necessity of a continuous glutamine supply for the sustenance of neurotransmission. Eventually, astrocytes release glutamine, which is then taken up by neurons and converted back into glutamate and GABA [79], forming a complete cycle.

### Astrocyte-neuron lactate shuttle (ANLS)

The concept of ANLS was first proposed in 1994, initially described as a mechanism that couples neuronal activity to glucose utilization [81]. The metabolic interaction is facilitated by a Na^+^-dependent uptake system rather than receptor-mediated processes [81]. Distinct from conventional anaerobic glycolysis, the ANLS model proposes that astrocytes take up glucose and convert it into lactate via aerobic glycolysis, which then serves as a significant energy substrate for neurons [2].

In the ANLS, *L*-lactate is released by astrocytes through transmembrane monocarboxylate transporter (MCT) 1 and 4 (MCT1 and MCT4). This process may involve high-capacity cation channels or pannexins. Through gap junctions, astrocytes establish a syncytium that allows for efficient distribution of *L*-lactate to active neurons [82]. *L*-lactate stimulates neuronal activity by undergoing transmembrane transport via MCT2 or by engaging specific receptors, such as the G protein-coupled receptor known as hydrocarboxylic acid receptor 1 (HCAR1). Once internalized into the neuronal cytoplasm via MCT2, *L*-lactate is oxidized into pyruvate by lactate dehydrogenase 1, a reaction that reduces NAD^+^ to NADH and releases H⁺ (L-lactate + NAD^+^ → pyruvate + NADH + H^+^). The resulting pyruvate enters the TCA cycle, while NADH feeds into the electron transport chain, which together support neuronal ATP production [83] (Fig. 1f).

In addition to supplying metabolic fuel to meet acute neuronal energy demands, the ANLS orchestrates downstream signaling cascades, thereby establishing a link between astrocyte-neuron metabolism and the shaping of behavior and cognition [84]. A central mediator of these effects is the G protein-coupled receptor HCAR1, which is expressed in both rodent and human brains and serves as a specific receptor for lactate [85]. Activation of HCAR1 by lactate triggers complex, and at times seemingly contradictory, physiological responses. The most well-characterized downstream effect of HCAR1 activation is the inhibition of adenylyl cyclase, which leads to reduced intracellular cAMP levels. This canonical signaling pathway has been linked to diverse functional outcomes, including the regulation of neuronal excitability. For instance, HCAR1 activation has been shown to reduce neuronal activity in cortical neurons [86, 87]. The effects of ANLS-related signaling, however, vary regionally, as evidenced by hippocampal slice studies showing that *L*-lactate and HCAR1 agonists enhance neuronal firing rates [88], while in the hypothalamic arcuate nucleus, lactate acts via astrocytic HCAR1 to depolarize pro-opiomelanocortin neurons [89]. These regional differences likely reflect variations in the HCAR1 expression patterns, the coupling to downstream effectors, or the cellular context.

Beyond the effects on neuronal excitability, HCAR1 signaling intersects with multiple molecular pathways that influence synaptic plasticity, neuroinflammation, and gene expression. At the synaptic level, lactate and an agonist of HCAR1 promote NMDAR-dependent synaptic and cognitive function by facilitating the co-agonist binding site occupancy of CA1 postsynaptic NMDARs [90]. This suggests a direct role for HCAR1 in gating synaptic plasticity. Concurrently, HCAR1 activation has been implicated in neurovascular coupling and may function as a volume transmitter in neuronal communication [91]. Lactate signaling through HCAR1 intersects with epigenetic regulation at the transcriptional level, where the lactate-derived histone lactylation links the cognitive benefits of daytime exercise to those of nighttime sleep [92], while HCAR1-mediated signaling influences neuroinflammatory tone [93]. During hypoglycemia, lactate acts through HCAR1 to protect axonal and myelin development [94].

The functional relevance of this signaling axis is further underscored by its involvement in complex behavior. Disruptions in astrocytic metabolism, such as those caused by activation of type 1 cannabinoid receptors, which impair lactate production, lead to altered neuronal function and deficits in social interaction behavior [95]. Similarly, mitochondrial Na^+^/Ca^2+^ exchange, which is highly enriched in astrocytes, shapes the cytoplasmic Ca^2+^ signaling and modulates glycolytic flux and lactate secretion in a Ca^2+^-dependent manner, thereby influencing cognitive outcomes [96]. Thus, the ANLS not only serves as an energy shuttle, but is also a conduit for astrocyte-derived signals. This provides a more comprehensive framework for understanding how astrocyte metabolic coupling contributes to brain functions and behavioral phenotypes.

### Mitochondrial ROS

Traditionally, mitochondrial ROS are regarded as detrimental byproducts of oxidative metabolism, closely linked to the pathological processes of ageing and neurodegenerative diseases. However, recent studies have strongly challenged this damage-centric framework, revealing the indispensable signaling roles of mitochondrial ROS in brain physiological functions. Astrocyte-derived mitochondrial ROS serve as key signaling molecules, supporting their specific metabolic programs, maintaining the neuron–astrocyte metabolic coupling, and ultimately influencing cognition and behavior.

The fundamental differences in energy metabolism between astrocytes and neurons lie in the distinct patterns of mitochondrial ROS production in these cells. Neurons primarily rely on OXPHOS to meet their energy demands, and their mitochondrial respiratory chain tends to assemble into highly efficient super complexes, a configuration that ensures efficient electron flow and energy production while limiting ROS generation [36, 53]. In contrast, astrocytes predominantly depend on glycolysis, and their mitochondrial respiratory chain exhibits a more loosely organized state, with complex I often existing in a free form [36, 97]. This structural feature results in less efficient OXPHOS in astrocytes but is accompanied by significantly higher mitochondrial ROS production in the physiological state [97]. Therefore, the high capacity for mitochondrial ROS generation in astrocytes is an intrinsic outcome of their specific metabolic configuration, rather than a manifestation of dysfunction [36, 98]. Interestingly, this OXPHOS-dependent metabolic pattern of neurons is complemented by their strategy of actively inhibiting glycolysis. By preserving nicotinamide adenine dinucleotide, neurons maintain healthy, energy-efficient mitochondria, which can subsequently utilize alternative fuels such as lactate and KBs provided by astrocytes to generate energy [99].

Studies have shown that astrocytes can utilize FA oxidation to sustain the inefficient, loosely organized conformation of their mitochondrial respiratory chain, thereby ensuring continuous production of signaling-competent mitochondrial ROS [53]. These mitochondrial ROS control the hypoxia-inducible factor cascade and the expression of key enzymes involved in glucose metabolism [95]. Furthermore, astrocytic mitochondrial ROS activate the nuclear factor erythroid 2-related factor 2 (Nrf2) pathway, upregulating a suite of antioxidant enzymes and finely modulating redox homeostasis within astrocytes [100]. More importantly, Nrf2 activation driven by astrocytic mitochondrial ROS promotes glutathione metabolism, enabling astrocytes to supply glutathione precursors to neurons. This is critical for neurons that have naturally weaker antioxidant defenses, since neuronal Nrf2 is highly unstable and struggles to cope with oxidative loads independently [100, 101]. Additionally, astrocyte-derived hydrogen peroxide can diffuse to adjacent neurons, thereby influencing neuronal synaptic function and plasticity [55].

Astrocytic mitochondrial ROS also impact whole-organism behavior. Studies have demonstrated that overexpressing mitochondria-targeted catalase in astrocytes to scavenge mitochondrial ROS impairs behavioral performance in mice [97]. Similarly, knockout of astrocytic carnitine palmitoyl transferase 1 A, which suppresses mitochondrial ROS production by altering respiratory chain assembly, leads to cognitive deficits in mice [53]. Moreover, astrocytic mitochondrial ROS mediate the behavioral regulation induced by extrinsic signals. For instance, activation of type 1 cannabinoid receptors on astrocytic mitochondria reduces phosphorylation of the complex I subunit NDUFS4, inhibiting complex I activity and mitochondrial ROS production. This signaling cascade diminishes lactate production via the hypoxia-inducible factor 1 pathway and ultimately impairs social interaction behavior in mice [95].

## Regional view of astrocyte-neuron metabolic coupling

**Fig. 2:**
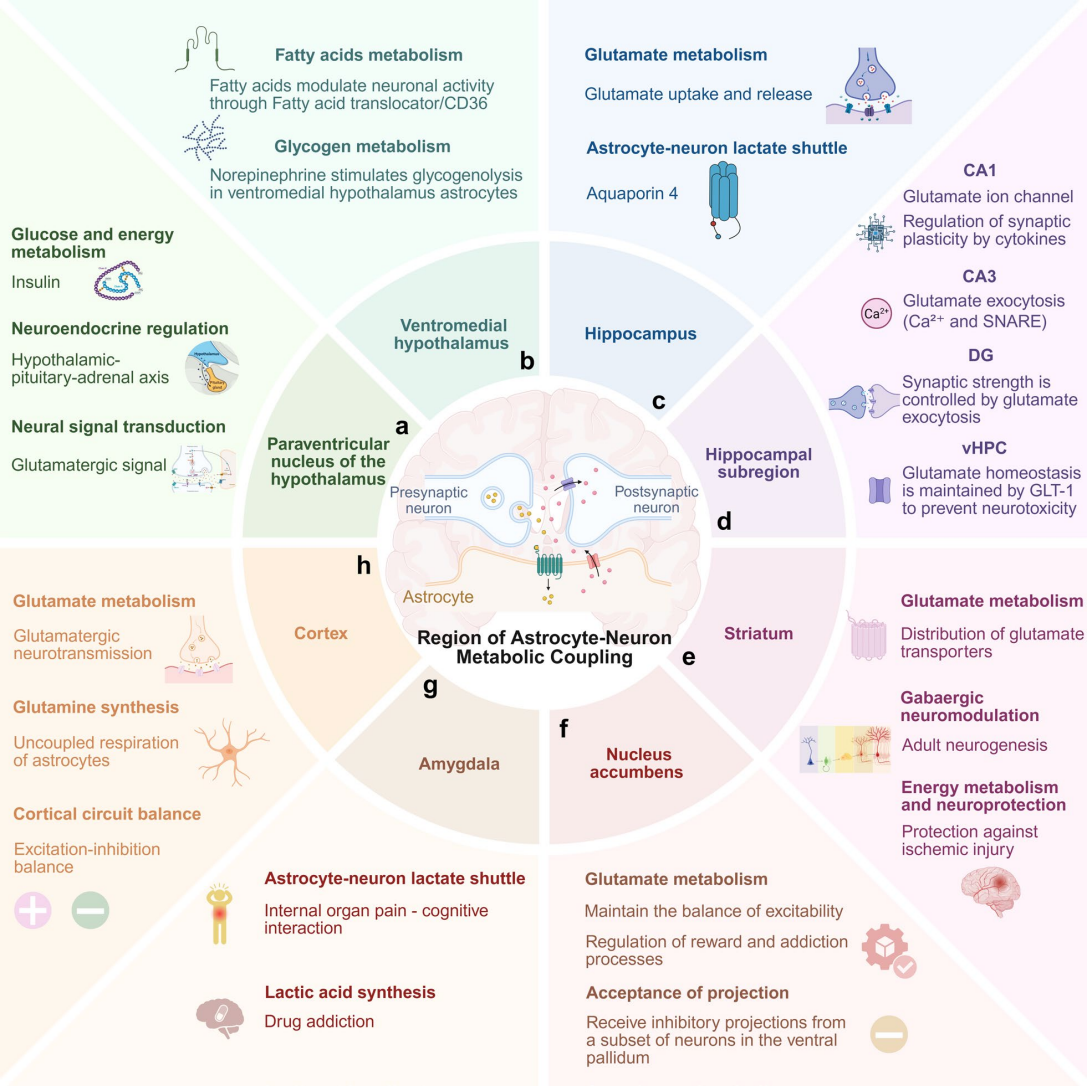
Astrocyte-neuron metabolic coupling in different brain regions. **a** Paraventricular nucleus of the hypothalamus. **b** Ventromedial hypothalamus. **c** Hippocampus. **d** Hippocampal subregion. **e** Striatum. **f** Nucleus accumbens. **g** Amygdala. **h** Cortex

The regional specificity of astrocyte-neuron metabolic coupling reflects an integration of molecular infrastructure, cellular state diversity, and functional demands. Astrocytes and neurons co-regulate neural functions through regional metabolic coupling mechanisms, with distinct brain regions exhibiting corresponding metabolic interaction patterns (Fig. 2) [2].

### Distribution of metabolite transporters and enzymes

The expression patterns of distinct metabolite transporters and metabolic enzymes constitute the principal axes of astrocyte-neuron coupling in different brain regions. In the hippocampus, the classical ANLS is dependent on the expression of aquaporin-4. Knockout of aquaporin-4 in mice significantly reduces the mRNA levels of glucose and lactate transporters in hippocampal astrocytes and neurons, leading to ANLS dysfunction and metabolic impairment [102].

Interestingly, in the striatum and NAc, the allocation of GLTs influences regional metabolism. Neuronal GLT-1 supplies glutamate to the synaptic TCA, and its specific knockdown instead increases astroglia-neuron glutamine transfer in the striatum [103]. This compensatory upregulation underscores the robustness of the regulation of the glutamine-glutamate cycle. In NAc, perturbations in the expression of GLTs in astrocytes disrupt glutamate homeostasis, highlighting their critical role in maintaining the balance of excitability, a mechanism directly related to addiction susceptibility [104].

In the cerebral cortex, the assignment of transporters follows a different logic. Studies using nuclear magnetic resonance spectroscopy have shown that a significant fraction of cortical energy expenditure is devoted to glutamatergic neurotransmission and that the glutamine-glutamate cycle is tightly coupled to glucose oxidation, with molar stoichiometric ratios close to 1:1 [105]. This quantitative relationship underscores the primacy of glutamate in cortical metabolism.

In the hypothalamus, FA transport emerges as a specialized feature. Within the ventromedial hypothalamus (VMH), FAs modulate neuronal activity through FA translocator/CD36 [106], reflecting the role of this region in integrating nutrient status and energy homeostasis.

### Metabolic demands generated by the activity of specific neural circuits

The specific activity of neural circuits in different brain regions shapes the patterns of different astrocyte-neuron metabolic coupling. The hippocampus, as the center of learning and memory, exhibits coupling mechanisms that are adapted to the demands of synaptic plasticity. Astrocytes detect neuronal activity via ion channels and neurotransmitter receptors, subsequently releasing gliotransmitters and synaptic proteins that modulate neurons [107, 108]. In the DG, glutamate exocytosis from astrocytes controls synaptic strength through neuronal activity-dependent stimulation of purinergic P2Y1 receptors on astrocytes [109]. At CA3-CA1 synapses, glutamate exocytosis depends on Ca^2+^ and SNARE protein activation in astrocytes, as well as metabotropic glutamate receptors in neurons [110]. Beyond vesicular exocytosis, CA1 astrocytes also directly release glutamate through glutamate-permeable ion channels [111, 112]. Additionally, the cytokine interleukin-33 is selectively regulated in hippocampal CA1 astrocytes to maintain homeostatic synaptic plasticity [113]. Astrocyte-derived tumor necrosis factor-α acts on postsynaptic tumor necrosis factor receptors to increase AMPA receptor density at excitatory synapses, thereby modulating synaptic strength and enabling neural circuits to adapt to activity changes [114]. These activity-dependent mechanisms ensure that metabolic support matches the synaptic demands induced by plasticity.

The striatum and NAc play an important role in the circuits mediating motor control and reward. Striatal GABAergic neurons are key to regulating adult neurogenesis. When their activity is photoinhibited, these neurons up-regulate fibroblast growth factor expression in endothelial cells. This astrocyte-mediated process may protect against oxygen–glucose deprivation and improve outcomes in ischemic brain injury [115]. The NAc serves as a critical hub for reward, pleasure, and addiction. Its strategic position within neural circuits allows it to integrate dopaminergic signals from the ventral tegmental area and glutamatergic inputs from the prefrontal cortex (PFC), ventral hippocampus, and BLA [116–118]. The glutamate metabolism within the NAc, particularly through neuron-astrocyte metabolic coupling, is central to the modulation of reward and addiction processes [119]. Specific neuron-astrocyte glutamatergic circuits are activated via mGluR5-mediated astrocyte Ca^2^⁺ signaling, underscoring the intricate cellular communication within the NAc [120]. The NAc also receives inhibitory projections from a subset of ventral globus pallidus neurons that promote reward-related behaviors [121].

There are interactions between hypothalamic astrocytes and neurons in the regulation of energy metabolism. In the paraventricular nucleus (PVN) of the hypothalamus, astrocytes regulate local neuronal activity and simultaneously influence glucose metabolism and energy homeostasis [6, 122]. Insulin serves as a central regulator in this process, altering cellular metabolic states by controlling the expression of genes related to glycolysis, the PPP, and lipid metabolism [123]. Experimental evidence shows that chemogenetic activation of PVN astrocytes significantly alters insulin sensitivity, thereby affecting peripheral glucose metabolism, providing novel insights into the mechanisms of insulin resistance [6]. Furthermore, PVN astrocytes participate in regulating the hypothalamic–pituitary–adrenal axis, a neuroendocrine pathway crucial for maintaining systemic glucose and energy metabolism balance [6]. Notably, these cells modulate the firing activity of PVN autonomic premotor neurons through glutamatergic signaling, subsequently influencing autonomic nervous system regulation of systemic metabolism [124, 125]. Additionally, astrocytes mediate the regulatory effects of angiotensin II on hypothalamic neuronal activity and sympathetic nervous system excitation [124]. Hypothalamic astrocytes also maintain mobilizable glycogen stores. Norepinephrine stimulates glycogenolysis in VMH astrocytes, thereby regulating the glucose-mediated signaling [126, 127]. This energy buffering capacity is particularly prominent in regions responsible for maintaining whole-body energy homeostasis.

The amygdala is a key component of the limbic system that plays a central role in contextual fear conditioning, which underlies emotional learning [128, 129]. Recent studies have highlighted the role of ANLS in promoting the synchronization of the BLA-anterior cingulate cortex network in rats, a phenomenon that is implicated in the interplay between visceral pain and cognitive processes [130]. Furthermore, lactate synthesis in the central amygdala has been implicated in drug addiction. Interestingly, disrupting the transport of lactate between astrocytes and neurons can mitigate cocaine seeking following extended periods of withdrawal [131, 132]. This suggests that metabolic coupling in the amygdala is not only involved in emotional events but also in long-term adaptation to addiction.

The cortex is required to maintain an excitation-inhibition balance while supporting plasticity. Disruption of this equilibrium can trigger a range of neurological disorders, including Parkinson’s disease (PD), epilepsy, and depression [2]. Acute GABA exposure does not stimulate glycolysis in cultured astrocytes but significantly enhances uncoupled respiration, a key metabolic effect that maintains glutamine synthesis in the mammalian cerebral cortex [133]. In the anterior cingulate cortex, activation of astrocytic Gi signaling reduces cyclic adenosine monophosphate and extracellular *L*-lactate levels. This reduction impairs the influx of *L*-lactate into neurons via MCT2, potentially compromising pattern memory and neuronal mitochondrial biogenesis [130].

## Dysregulations of astrocyte-neuron metabolic coupling in ageing-related CNS diseases and potential therapeutic strategies

Regional metabolisms are not static but evolve with age. Age-related stressors, such as mitochondrial dysfunction and oxidative stress, can differentially impair these couplings across brain regions, driving the regional pathology of age-associated brain diseases. Consequently, targeting key nodes within these metabolic pathways represents a promising strategy to counteract the underlying age-related metabolic decline. Interventions from repurposed drugs to novel agents have the potential to restore metabolic resilience (Table 1) [2].

**Table 1:** Drugs and their targets involved in astrocyte-neuron coupling metabolism

Drug name	Target	Mechanism	Primary target cell	Metabolism related to astrocyte-neuron coupling	Other affected cells	Potential off-target effects	Disease	Research stage	Availability
Bexarotene	ApoE [203]	Increases ApoE levels in astrocytes to modulate cerebral Aβ accumulation and clearance	Astrocytes	Lipid metabolism	Microglia, neurons	May affect microglial phagocytosis [204] and neuronal lipid homeostasis	AD	Preclinical research	Experimental
Bezafibrate	*Acadvl* [152]	Activates FAO in astrocytes, promotes FA decomposition, prevents LD accumulation, alleviates neuroinflammation, and improves synaptic function	Astrocytes	Lipid metabolism	Microglia, neurons	PPAR pan-agonist may affect multiple cell types; potential systemic metabolic effects [205]	AD	Preclinical research	Experimental
Fenofibrate	*Pparα* [152]	Activates FAO in astrocytes, promotes FA decomposition, prevents LD accumulation, alleviates neuroinflammation, and improves synaptic function	Astrocytes		Microglia, neurons, hepatocytes	Systemic PPARα activation affects liver metabolism; may alter systemic lipid profiles [206]		Phase II clinical trial	Experimental
*Cpt2* agonist	*Cpt2*	Activates FAO in astrocytes, promotes FA decomposition, prevents LD accumulation, alleviates neuroinflammation, and improves synaptic function	Astrocytes		Neurons, microglia	May affect neuronal mitochondrial function		Preclinical research	Experimental
DPCPX	A1R [145]	Inhibits the A1R-Lcn2 signaling pathway, suppresses astrocyte activation and improves memory deficits	Astrocytes	Lipid metabolism	Neurons, microglia	A1R widely expressed in CNS; may affect neuronal excitability and synaptic transmission [207]	AD	Preclinical research	Experimental
LDHB inhibitor	LDHB [151]	Reduces lactate production and blocks excessive lactate transfer from astrocytes to neurons, mitigates neuronal toxicity	Neurons	Glucose metabolism + ANLS	Astrocytes	May impair physiological lactate utilization in neurons	AD	Preclinical research	Experimental
Memantine [208] (Fig. 3b)	NMDAR [148]	Reduces Ca^2+^ overload, enhances astrocytic glucose catabolism, improves TCA cycle efficiency, and restores cerebral energy supply	Neurons	Glucose metabolism	Astrocytes	May affect synaptic plasticity and excitotoxicity balance [209]	AD	Phase IV clinical trial	On the market
PKM inhibitor	PKM [151]	Reduces glycolysis, forces astrocytes to shift toward OXPHOS, thereby improving energy metabolism	Astrocytes (PKM2), all cells (PKM1)	Glucose metabolism + glutamate/GABA-glutamine cycle	All glycolytic cells (neurons, microglia, oligodendrocytes)	May impair physiological glycolysis in all cell types; risk of systemic metabolic disruption	AD	Preclinical research	Experimental
Sodium butyrate (Fig. 3d)	SCFA [210]	Modulates GS activity to rescue glutamate trafficking deficits in astrocyte-neuron coupling, thereby enhancing the glutamate-glutamine shuttle to protect neurons from oxidative damage	Astrocytes	Glutamate/GABA-glutamine cycle	All cell types	Broad HDAC inhibitor affects gene expression in all CNS cells [211]	AD	Preclinical research	Experimental
Syrosingopine	MCT [152]	Reduces lactate efflux from astrocytes, alleviates extracellular acidosis and neuronal injury, while forcing astrocytes to shift toward OXPHOS, improving their own energy metabolism	Astrocytes (MCT4)	Glucose metabolism + ANLS	Oligodendrocytes (MCT1), neurons (MCT2)	May compromise myelin maintenance [212]	AD	Preclinical research	Withdrawal from the market
A485 (Fig. 3h)	H3K9la [213]	Reduces H3K9la levels, blocks lactylation catalytic enzymes, prevents excess lactate shuttling from astrocytes to neurons, thus decreasing oxidative stress	All cells	Glucose metabolism + ANLS	All CNS cell types	Broad epigenetic effects; may alter gene expression programs in all cells [214]	PD	Preclinical research	Experimental
DNL201 (Fig. 3i)	LRRK2 [154]	Restores astrocytic glutamate uptake via EAAT1/2, attenuates excitotoxicity; restores the pathway of the ANLS for dopaminergic neuroprotection	Astrocytes, neurons	Glucose metabolism + ANLS + glutamate/GABA-glutamine cycle + lipid metabolism	Microglia	May affect microglial function and neuronal vesicle trafficking	PD	Preclinical research	Experimental
Probucol (Fig. 3g)	ApoE [203]	Increases ApoE levels in astrocytes to modulate cerebral Aβ accumulation and clearance	Astrocytes	Lipid metabolism	Microglia, neurons, hepatocytes	Cholesterol-lowering agent with systemic effects [215]; may alter lipoprotein metabolism	PD	Phase II clinical trial	Withdrawal from the market
Terazosin	PGK1 [216]	Enhances glycolytic activity in astrocytes to provide energetic support to neurons	All cells	Glucose metabolism	All glycolytic cells	α1-adrenergic receptor antagonist; may affect vascular tone and blood pressure	PD	Preclinical research	On the market
Bezafibrate; L-Carnitine	FAO [8]	Inhibits astrocytic conversion of glucose to FA, reduces energy production and decreases ROS generation, thereby mitigating neuronal damage	Astrocytes	Lipid metabolism	Hepatocytes, cardiomyocytes, neurons	PPAR pan-agonist with systemic metabolic effects; may alter whole-body lipid metabolism [205]	HD	Preclinical research	On the market
Dapagliflozin	SGLT2 [168]	Suppresses neuronal glucose uptake from astrocytes, decreases glycolytic reliance, lowers lactate generation, alleviates acidotoxicity and oxidative stress	Renal tubules	Glucose metabolism + ANLS	Neurons	Main effect may be systemic glucose lowering [217]	HD	Preclinical research	On the market
Metformin (Fig. 3e)	AMPK [218]	Modulates astrocytic aerobic glycolysis, particularly glycogen turnover and the shuttle of glucose- and glycogen-derived lactate from astrocytes to neurons, maintains cellular energy homeostasis	All cells	Glucose metabolism + ANLS	All CNS cell types	Broad AMPK activator with systemic metabolic effects; may affect whole-body energy homeostasis	HD	Preclinical research	On the market
ALKBH5 inhibitor (Fig. 4b)	ALKBH5 [219]	Regulates m6A modification on GLT-1, enhances its expression in astrocytes, thereby rescuing stress-induced disruptions in glutamatergic synaptic transmission, neuronal atrophy, and impaired Ca^2+^ activity	All cells	Glutamate/GABA-glutamine cycle	All CNS cell types	Broad m6A modification changes; may alter RNA metabolism in all cells [220]	Depression	Preclinical research	Experimental
Fluoxetine (Fig. 4a)	GR [221]	Enhances astrocytic glucose uptake and glycolysis under corticosterone-induced depression by inhibiting the GR-TXNIP-GLUT1 pathway	Neurons	Glucose metabolism	Astrocytes, all 5-HT-responsive cells	SSRIs primarily target neuronal serotonin reuptake; astrocyte effects may be indirect	Depression	Phase IV clinical trial	On the market
Montelukast	CysLT_1_R [222]	Upregulates GLT-1 expression in astrocytes, reduces synaptic glutamate concentrations, ameliorates depressive phenotypes through modulating glutamatergic neurotransmission	Astrocytes	Glutamate/GABA-glutamine cycle	Microglia, peripheral immune cells	May affect peripheral and central inflammation	Depression	Phase II clinical trial	On the market
Arundic acid (Fig. 4e)	S100B [223]	Restores MCT1/4 function, rescues lactate metabolic support, thereby alleviating neuronal energy deficits. Mitigates the inhibition of astrocytic GS, restoring the glutamate-glutamine cycle	Astrocytes	Glucose metabolism + ANLS + g lutamate/GABA-glutamine cycle	Neurons, microglia	S100B also affects microglial activation; may modulate neuroinflammation [224]	SCZ	Preclinical research	Experimental
miR-137 antagomir	miR137 [225]	Upregulates *LDHA*, enhances astrocytic glycolytic lactate production, thereby supporting neuronal energy demands. Alleviates GLUT1 inhibition, improves astrocytic glucose uptake, reinforces metabolic support for neurons	All cells	Glucose metabolism + ANLS	All CNS cell types	miR-137 regulates broad gene expression programs; may affect neuronal development and synaptic function [226]	SCZ	Preclinical research	Experimental

ANLS, Astrocyte-neuron lactate shuttle; EAAT, excitatory amino acid transporter; GS, glutamine synthetase; LD, lipid droplet; L lactate dehydrogenase B-chain; MCT, monocarboxylate transporter; PKM, pyruvate kinase M; SCFA, FAO, fatty acid oxidation

### Astrocyte-neuron metabolic coupling in non-pathological ageing

In the non-pathological ageing brain, astrocytes exhibit a gradual reduction in metabolic function. Studies have demonstrated that aged astrocytes show decreased glucose uptake, reduced GS activity, and lower GLUT1 expression levels, accompanied by a compensatory upregulation of glutathione levels and antioxidant systems [134]. This decline in basal metabolic capacity is further reflected in altered glycogen metabolism, with accumulation of under-branched glycogen aggregates in the hippocampus, suggesting impaired energy storage dynamics even when cognitive function remains preserved [135].

Astrocytic mitochondria undergo significant changes with age, characterized by increased ROS production and enhanced sensitivity to inflammatory stimuli [136, 137]. These age-related stressors, including DNA damage responses, oxidative stress, and mitochondrial dysfunction, lead to impaired metabolic coupling between astrocytes and neurons. The resulting decline in synaptic plasticity directly contributes to age-associated cognitive decline [138, 139]. Consistent with this, astrocytic senescence has emerged as a key mediator of brain ageing, with senescent astrocytes displaying characteristic markers including SA-β-gal activity, p16INK4a upregulation, and secretion of senescence-associated secretory phenotype factors that disrupt local metabolic homeostasis [139].

Importantly, brain ageing is not a uniform process, but exhibits marked regional heterogeneity [22, 140]. This regional vulnerability involves differential suppression of GLUT4 and ApoE expression, two key proteins mediating astrocyte-directed glucose and lipid metabolism [141]. This regional heterogeneity in metabolic decline may underlie the selective susceptibility of areas like the hippocampus and substantia nigra to age-related neurodegeneration [142]. Intriguingly, MCT2 has emerged as a potential compensatory factor that can independently facilitate neuronal energy uptake, suggesting that the astrocyte-neuron metabolic coupling retains adaptive capacity even in the ageing brain [141]. This preserved compensatory mechanism may delay the transition from physiological ageing to pathological decline.

### Neurodegenerative diseases

#### AD

AD is one of the most prevalent age-associated forms of dementia, characterized by Aβ accumulation and tau hyperphosphorylation. The early pathological changes predominantly occur in the hippocampus, a region particularly vulnerable to age-related metabolic stress [143, 144]. In AD model mice, neuronal expression of adenosine receptor 1 (A1R) is significantly upregulated in a tau pathology-dependent manner [145]. Mechanistic studies revealed that A1R activation in neurons promotes the release of lipocalin-2 (Lcn2), which subsequently triggers astrocytic activation. Importantly, neuron-specific silencing of Lcn2 not only effectively suppressed astrocyte activation but also significantly improved synaptic plasticity and restored learning/memory functions in mice. These findings demonstrate the critical role of the A1R-Lcn2 signaling pathway in AD pathogenesis and suggest that tau pathology remodels the neuron-glia lipid metabolic crosstalk and accelerates neurodegenerative progression [145]. Furthermore, recent studies have revealed dysfunction in astrocyte-neuron interactions through the glutamate-glutamine cycle during tau-dependent neurodegeneration [146], further validating that the metabolic crosstalk between astrocytes and neurons constitutes a crucial component of AD pathogenesis.

**Fig. 3:**
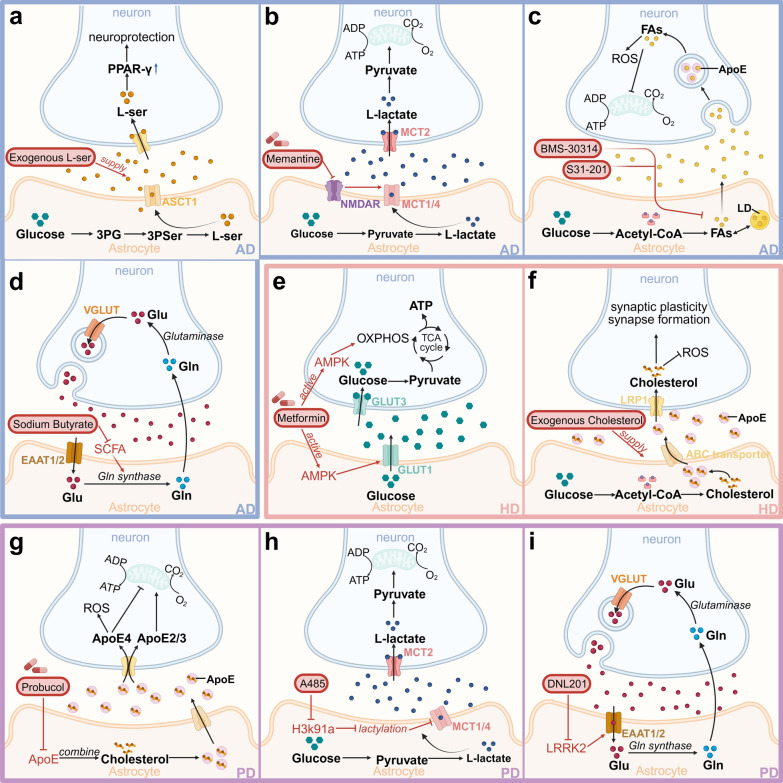
Neurodegenerative disease therapeutics and their corresponding targets in the astrocyte-neuron metabolic coupling pathway. **a** Exogenous *L*-Ser supplementation alleviates AD symptoms and exerts neuroprotective effects. **b** Memantine enhances lactate release in the ANLS via NMDAR inhibition, offering therapeutic potential for AD. **c** BMS-30314 and S31-201 attenuate oxidative stress and neuroinflammation in AD by targeted inhibition of FA metabolism. **d** Sodium butyrate targets SCFA metabolism, upregulates glutamine synthetase, and enhances glutamine synthesis for AD therapy. **e** Metformin activates AMPK, upregulates GLUT1 and glycolysis to enhance cerebral energy supply, offering therapeutic potential for HD. **f** Exogenous cholesterol supplementation enhances synaptic plasticity and remodeling, offering therapeutic benefits for HD. **g** Probucol targets ApoE, inhibiting ApoE4-induced oxidative stress and mitochondrial damage for PD treatment. **h** A485 targets H3K91a to block lactylation catalytic enzymes, suppress excessive lactate shuttle and mitigate oxidative stress for PD treatment. **i** DNL201 targets LRRK2 to restore astrocytic glutamate uptake via EAAT1/2 for PD treatment. AD: Alzheimer’s disease; ANLS: Astrocyte-neuron lactate shuttle; EAAT: Excitatory amino acid transporter; FAs: Fatty acids; Gln: Glutamine; Glu: Glutamate; GLUT: Glucose transporter; HD: Huntington’s disease; LD: Lipid droplet; *L*-Ser: *L*-Serine; OXPHOS: Oxidative phosphorylation; PD: Parkinson’s disease; SCFA: Short-chain fatty acid; VGLUT: Vesicular glutamate transporter

Based on previous studies, methylglyoxal is a cytotoxic and mutagenic byproduct of coupled glucose, lipid, and amino acid metabolism in astrocytes and neurons. The neurotoxicity observed in AD may be associated with methylglyoxal overproduction resulting from enhanced aerobic glycolysis, coupled with limited detoxification capacity in brain regions most vulnerable to AD pathogenesis, including the entorhinal cortex, precuneus, and posterior cingulate cortex [147]. These findings support the feasibility of developing novel AD therapeutics targeting methylglyoxal. In addition, AD mice exhibit synaptic and behavioral deficits upon inactivation of the *L*-Ser biosynthesis pathway in hippocampal astrocytes, whereas dietary *L*-Ser supplementation prevents these impairments (Fig. 3a), suggesting that oral *L*-Ser administration represents a ready-to-use therapeutic strategy for AD [148]. *APOE4* is the most significant genetic risk factor for cognitive impairment and AD. Data from a study of Chinese adults aged 80 and older have substantiated the link between *APOE4* with both ageing and AD [149]. Consequently, *APOE4* has emerged as an effective therapeutic target and a central focus for targeted drug development in AD [150].

The elevated levels of astrocytic metabolic proteins in the cerebrospinal fluid of AD patients further underscore the pivotal role of astrocyte metabolism in AD pathogenesis [151]. Moreover, these proteins, including PKM and LDHB (lactate dehydrogenase B-chain), may serve as robust biomarkers for both staging AD progression and developing novel therapeutic interventions for this disorder. A study demonstrated that Tfam deficiency-induced OXPHOS impairment triggers LD accumulation and exhibits prominent AD-like pathology. Additionally, pretreatment with either the ATP-citrate lyase (ACLY) inhibitor BMS-303141 or the STAT3 inhibitor S3I-201 (Fig. 3c) attenuated the FA overload-induced astrocyte reactivity, a process closely linked to AD pathogenesis [152]. These findings suggest that Tfam, ACLY, and STAT3, which are key regulators of astrocyte-neuron coupling metabolism, represent potential therapeutic targets for AD.

#### PD

PD is the second most common age-associated neurodegenerative disorder. The pathological hallmarks of PD include loss of dopaminergic neurons and the presence of aggregated α-synuclein, primarily localized in the substantia nigra pars compacta of the midbrain [153]. Astrocytes critically contribute to PD pathogenesis by modulating dopaminergic neuronal death through their roles in glutamate-mediated excitotoxicity, K^+^ buffering, and Ca^2+^ homeostasis. Age-compromised capacity of astrocytes to handle excitotoxicity and maintain metabolic balance contributes to disease progression [8]. Furthermore, dysfunction in lipid metabolic pathways between astrocytes and dopaminergic neurons is consistently observed in PD [154]. These findings collectively underscore that the progression of PD is fundamentally linked to the breakdown of age-sensitive astrocyte-neuron metabolic coupling. Previous studies have identified energy metabolism disorders in the striatum and substantia nigra pars compacta as a primary cause of PD-like neurodegeneration, characterized by decreased ATP levels and reduced expression of genes and proteins associated with ATP production, which is closely related to glucose metabolism [155]. While a substantial body of research has focused on the association between ApoE and AD, a seminal study from 2012 provided the first evidence of ApoE receptor activation in a mouse model of PD, highlighting the link between altered lipid metabolism and neurodegeneration [156]. This finding underscores the importance of ApoE in the pathogenesis of PD, implying substantial therapeutic potential of ApoE-targeted drug development for PD treatment.

Key nodes within the ANLS pathway have emerged as potential hotspots for targeted therapeutic intervention. Under physiological conditions, activity-dependent lactate flux is essential for maintaining neuronal energy homeostasis, redox balance, synaptic plasticity, and signaling through receptors such as HCAR1 [84, 90]. However, under pathological conditions, particularly in the context of mitochondrial dysfunction or impaired lactate clearance mechanisms, the balance between lactate production and utilization is disrupted. This shift transforms lactate from a supportive metabolite into a pathologically accumulated metabolite that may exacerbate neurodegeneration [157]. Accordingly, therapeutic strategies targeting lactate metabolism may exert stage-dependent effects [158]. In early-stage disease, where lactate flux itself is primarily compromised, interventions should focus on restoring astrocytic glycolytic function to meet neuronal energy demands. Conversely, in late-stage disease characterized by pathological lactate accumulation that neurons cannot effectively utilize, the therapeutic priority should shift toward promoting lactate clearance or inhibiting its aberrant production.

Cerebrospinal fluid lactate levels in PD patients exhibit a biphasic pattern. In early-stage PD, lactate levels are decreased [159], whereas in late-onset PD patients, cerebrospinal fluid lactate levels are abnormally elevated [83]. This dynamic shift suggests that lactate may play distinct roles at different stages of PD.

In early-stage PD, when lactate levels are low, physiological lactate may exert neuroprotective effects. Exposure of cultured SH-SY5Y cells, primary neurons, or astrocytes to lactate-containing medium after MPP^+^ treatment, induces mitophagy and autophagy, thereby restoring mitochondrial activity and protecting cells from necrosis and apoptosis [160]. Similar effects have been observed in human fibroblasts carrying PD-associated mutations following lactate exposure [161]. These findings indicate that maintaining appropriate physiological levels of lactate in early disease stages may confer neuroprotection through supporting mitochondrial function.

In late-stage PD, however, as the disease progresses and mitochondrial function becomes severely compromised, dopaminergic neurons upregulate compensatory glycolysis, leading to increased lactate generation in an attempt to maintain ATP levels. This compensatory mechanism, however, promotes dopaminergic neuronal apoptosis via accumulation of pathological lactate. In this context, inhibition of hexokinase 2 expression attenuates pathological lactate production via the AMPK/Akt/mTOR pathway, thereby improving motor behavior and reducing dopaminergic neuronal loss [157]. Another PD study reached consistent conclusions and proposed that sirtuin 1 regulates cerebral lactate homeostasis through deacetylation and inhibition of PKM2, thereby mitigating PD pathology [162]. Together, these studies suggest that in late-stage PD, suppressing pathological lactate accumulation may represent a more effective therapeutic strategy.

In summary, the ANLS provides potential molecular targets for PD therapy, but the selection of intervention strategies must consider disease stage.

#### HD

HD is an autosomal-dominant neurodegenerative disorder caused by mutations in the huntingtin (*HTT*) gene. While genetically determined, its onset and progression are significantly modulated by ageing, with the age-dependent accumulation of cellular damage interacting with mutant *HTT* toxicity [163]. HD is characterized by predominant pathological changes in the basal ganglia circuitry, particularly affecting GABAergic projection neurons in the striatum [163]. Research has revealed significant regional vulnerability to mutant *HTT*, demonstrating a hierarchical susceptibility pattern: striatum > cortex>> hippocampus >>> midbrain, confirming the central role of striatum in HD pathogenesis [164]. Current evidence suggests that HD pathology results from the combined effects of CAG repeat expansion and basal ganglia circuitry impairment [163].

In HD, astrocytes express abundant levels of mutant *HTT*, whose accumulation disrupts glutamate uptake, K^+^ homeostasis, Ca^2+^ signaling, and metabolic reprogramming, leading to astrocytic dysfunction and exacerbating neurodegeneration. Studies have shown downregulation of EAAT2 in HD astrocytes, causing glutamate dyshomeostasis and subsequent disruption of the astrocyte-neuron glutamate/GABA-glutamine cycle [8]. For instance, in R6/2 transgenic mouse models, reduced glutamine synthesis and release in the striatum directly impairs neuronal GABA synthesis, indicating that the dysfunctional astrocyte-neuron metabolic coupling represents a critical pathogenic mechanism in HD [164].

Furthermore, HD patients exhibit significant lipid metabolism disturbances, including reduced brain cholesterol levels. Experimental studies demonstrate that cholesterol (Fig. 3f) or ganglioside GM1 supplementation improves cognitive and motor functions in HD mice, reduces mutant HTT aggregation, and restores synaptic function, suggesting the therapeutic potential of targeting lipid metabolism [165, 166]. Glucose metabolism abnormalities are also prominent in HD. Deficient expression of GLUT-1 and GLUT-3 in juvenile HD patients potentially compromises neuronal energy supply [167]. Recent studies indicate that sodium-glucose cotransporter-2 inhibitors may alleviate HD symptoms by improving astrocyte-mediated glucose metabolism [168]. These findings provide novel therapeutic directions targeting astrocyte-neuron metabolic coupling, warranting further investigation for clinical translation.

In addition, it is worth noting that astrocytes primarily provide metabolic support to neurons through glycolysis and lactate release. PFKFB3 is a key regulator of this process in the brain. Under physiological conditions, astrocytes exhibit relatively high PFKFB3 abundance because APC/C-Cdh1 activity is low in astrocytes; by contrast, in differentiated neurons, APC/C-Cdh1 continuously polyubiquitinates PFKFB3 and targets it for proteasomal degradation, thereby limiting neuronal glycolysis and allowing glucose-6-phosphate to be preferentially metabolized through the PPP to sustain antioxidant defense [35, 37]. Accordingly, pathological stabilization/accumulation of PFKFB3 in neurons, triggered by excitotoxic NMDAR signaling [169], lysosomal dysfunction and mitochondrial ROS in CLN7 (ceroid lipofuscinosis, neuronal 7) [170], inflammatory proteasome remodeling in multiple sclerosis [171, 172], or forced neuronal PFKFB3 expression *in* *vivo* [37], drives a metabolic switch from PPP to glycolysis, NADPH/GSH depletion, mitochondrial/redox stress and neuronal death. Therefore, the genetic or pharmacological modulation of PFKFB3 must be interpreted in a cell-type and context-dependent manner: targeting aberrant neuronal PFKFB3 stabilization is neuroprotective, whereas the astrocytic PFKFB3 supports glycolysis which underlies the physiological neuron-glia metabolic coupling.

However, the role of fructose-2,6-bisphosphate (F2,6BP), a product of PFKFB3, must be considered within the context of specific pathological stages and cell types. A study on HD reported that direct supplementation of F2,6BP in cell and *Drosophila* models of HD restored genomic integrity and alleviated disease symptoms [173, 174], suggesting that F2,6BP supplementation may represent a promising therapeutic strategy. Importantly, this should not be conflated with chronic neuronal PFKFB3 stabilization, which consistently suppresses PPP activity and promotes oxidative injury and neurodegeneration [35, 37, 172]. Thus, a “time window” concept for metabolic interventions is plausible. An explicit distinction between acute metabolic support in energy-depleted settings versus pathological, sustained neuronal glycolytic activation via PFKFB3 accumulation is needed.

### Psychiatric disorders

#### Depression

The pathogenesis of depression involves complex neurobiological alterations, with multisystem interactions at its core. As an age-associated psychiatric disorder, these interactions are significantly amplified within the ageing context. Age-related dysregulation of the hypothalamic–pituitary–adrenal axis and diminished stress resilience exacerbate neuroendocrine dysfunction, thereby intensifying stress-linked structural brain damage [175]. Under chronic stress, key emotion-regulating brain regions, including the medial PFC, hippocampus, and amygdala, exhibit marked age-related neuronal atrophy and synaptic loss, establishing persistent negative emotional circuitry [176, 177]. At the neurotransmitter level, dysfunction in serotonin, norepinephrine, and dopamine systems directly impairs emotional regulation circuits, motivation processing, and cognitive function [178]. Concurrently, dysregulation of the glutamate/GABA–glutamine cycle plays a more immediate role in depression. Impaired function of GLT-1 in the PFC leads to extracellular glutamate accumulation, while deficits in GABAergic signaling disrupt the precise neural activity modulation [179, 180]. These cumulative changes, driven by ageing and disease processes, can trigger significant emotional and behavioral abnormalities [181].

Recent research highlights the central regulatory role of astrocytes in depression. Vesicular nucleotide transporter expression is downregulated in depression. As this protein facilitates ATP loading into secretory vesicles, its dysfunction further compromises astrocytic energy metabolism regulation [182]. In the medial PFC, glucocorticoid receptors modulate astrocytic calcium dynamics and ATP release, suppressing glycolysis and reducing lactate production. This impairs the excitability of layer V pyramidal neurons [183, 184]. Experimental evidence shows that peripheral lactate supplementation can reverse the neuronal excitability deficits, offering novel therapeutic potential [185]. Under conditions of insufficient lactate support, lactate supplementation may restore metabolic coupling, provide energy to neurons, and exert neuroprotective effects. This aligns with the disease-stage dependency of lactate-targeted therapies discussed above.

**Fig. 4:**
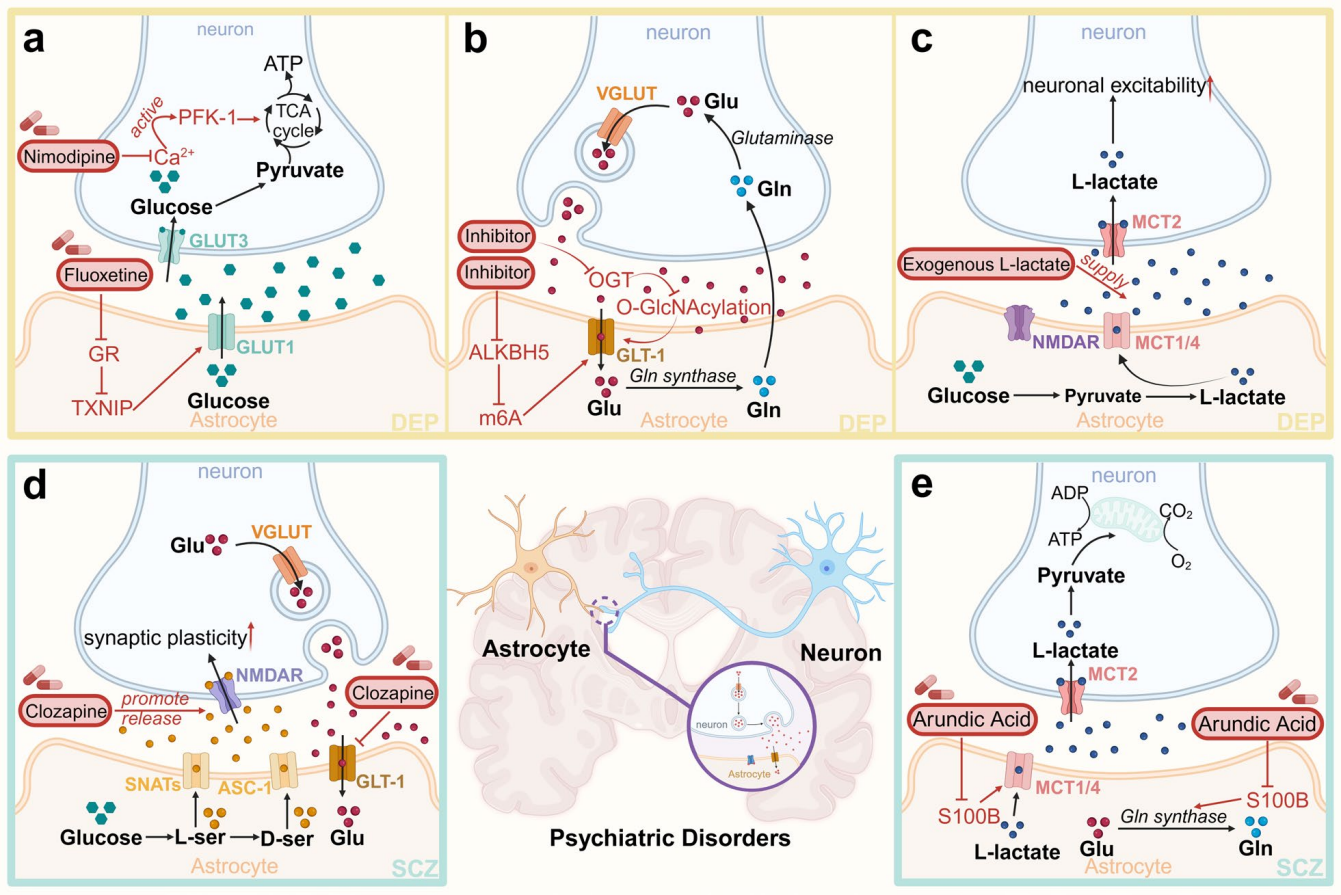
Psychiatric disorder therapeutics and their corresponding targets in the astrocyte-neuron metabolic coupling pathway. **a** Fluoxetine limits GR nuclear translocation, thereby suppressing TXNIP expression. The subsequent reduction in TXNIP levels relieves the inhibition of GLUT1, restoring glucose uptake and glycolytic flux in astrocytes. Nimodipine inhibits Ca^2+^ channels to activate PFK-1, thereby enhancing glycolysis for depression treatment. **b** ALKBH5 and OGT inhibitors enhance glutamate uptake by suppressing m6A demethylation and *O*-GlcNAcylation respectively, offering a therapeutic strategy for depression. Exogenous lactate supplementation enhances neuronal excitability and exhibits therapeutic potential for depression. **d** Clozapine modulates glutamatergic neurotransmission in SCZ by inhibiting GLT-1 to reduce glutamate uptake, enhancing synaptic glutamate availability, while concurrently promoting *D*-Ser and *L*-Ser release. **e** Arundic acid targets S100B to enhance lactate transport and glutamate synthesis, offering therapeutic potential for SCZ. DEP: Depression; *D*-Ser: *D*-Serine; EAAT: Excitatory amino acid transporter; GR: glucocorticoid receptor; Gln: Glutamine; Glu: Glutamate; GLUT: Glucose transporter; *L*-Ser: *L*-Serine; MCT: Monocarboxylate transporter; SCZ: Schizophrenia; TXNIP: Thioredoxin-interacting protein; VGLUT: Vesicular glutamate transporter

At the molecular level, new treatment targets have been identified. Studies revealed that astrocytes in the lateral habenula (LHb) mediate secondary-wave neuronal activation via glutamate and ATP/adenosine. Activating or inhibiting the LHb astrocytic Ca^2+^ signaling induces or prevents stress-induced depressive behaviors, respectively (Fig. 4a) [186]. Additionally, the *O*-GlcNAc transferase in medial PFC astrocytes is involved in depressive-like behavior by modulating glutamatergic synaptic transmission through *O*-GlcNAcylation of GLT-1, and the GABA_A_ receptor has been validated as a potential target for rapid antidepressant effects (Fig. 4b) [181, 187].

#### Schizophrenia (SCZ)

SCZ is a severe psychiatric disorder with high clinical heterogeneity, characterized by neuropathological alterations involving structural and functional abnormalities in multiple key brain regions, often following distinct, accelerated trajectories of ageing. Notably, pathological changes in the dorsolateral PFC play a central role in the cognitive dysfunction in SCZ, with neuronal microcircuit abnormalities being closely associated with core symptoms such as working memory deficits [188]. Further research has revealed that dysintegration among large-scale neural networks at the whole-brain level exacerbates the impairments of cognitive and emotional processing, a systemic dysfunction likely rooted in the disturbance of multiple neurotransmitter systems [189] and often compounded by premature cellular ageing and age-associated comorbidities [190].

In-depth studies demonstrated that SCZ is characterized by a dual neurochemical dysregulation, involving dopaminergic hyperactivity and glutamatergic NMDAR hypofunction. These neurotransmitter abnormalities not only directly inhibit the function of GABAergic interneurons but also disrupt cortical information processing synchronization [191]. Concurrently, widespread impairments in synaptic plasticity not only affect the efficiency of neuronal signaling but may also constitute a key pathological foundation for disease progression. Recent transcriptomic studies have identified marked abnormalities in lipid metabolism pathways in neurons and astrocytes of SCZ patients, particularly the downregulation of genes related to FA metabolism and cholesterol biosynthesis [11].

At the metabolic level, dysfunction of the ANLS system interferes with the supply of energy substrates, thereby affecting higher-order cognitive functions [192]. More specifically, defects in post-translational modification of GLAST and GLT-1 not only disrupt glutamate homeostasis, but also play a critical role in disease pathogenesis as validated in mouse models [193]. Regarding therapeutic mechanisms, clozapine, a highly effective drug for treatment-resistant SCZ, stands out due to its unique ability to modulate multiple aspects of astrocyte-neuron metabolic coupling. This drug effectively ameliorates NMDAR hypofunction by regulating GLT expression and promoting gliotransmitter release (Fig. 4d) [194]. Additionally, abnormalities in the *D*-Ser metabolic pathway and functional deficits in its synthetic enzymes provide important clues for developing novel targeted therapies [195].

## Conclusions

**Fig. 5:**
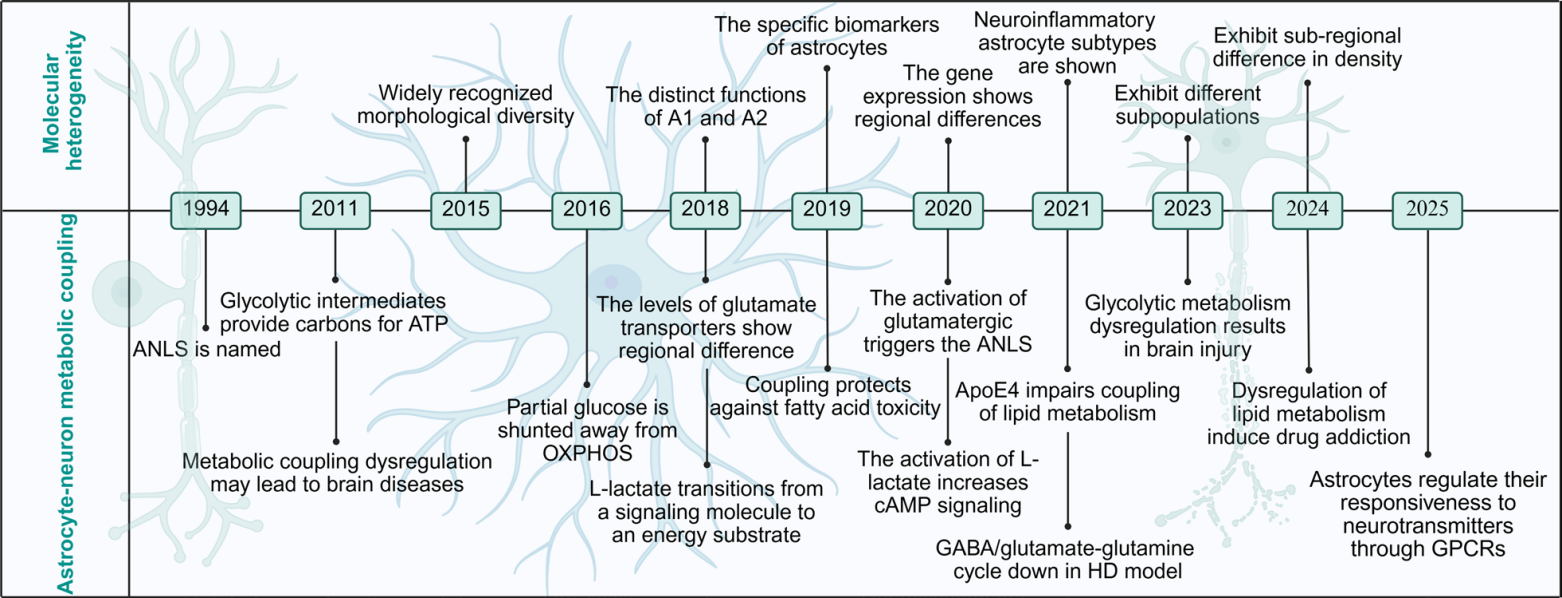
Research milestone of astrocyte molecular diversity and astrocyte-neuron metabolic coupling characteristics. Astrocytes were historically viewed as a homogeneous group. A paradigm shift occurred after the subtypes were found. This pivotal discovery catalyzed a surge in research aimed at identifying specific biomarkers for these astrocyte subtypes, an endeavor that has been persistently pursued over the years. Continuing this trajectory, a study brought to light the molecular heterogeneity of astrocytes across diverse brain regions. The reality was that these specialized subtypes are not only present under normal conditions but also manifest in pathological states. Most notably, studies emphasize the remarkable heterogeneity of astrocytes even within subregions of the same brain area. A1: Astrocyte1; A2: Astrocyte2; ANLS: Astrocyte-neuron lactate shuttle; ApoE4: Apolipoprotein E4; cAMP: Cyclic adenosine monophosphate; GABA: γ-Aminobutyric acid; GPCRs: G protein-coupled receptors; HD: Huntington’s disease; OXPHOS: Oxidative phosphorylation

Astrocytes are the most abundant non-neuronal cells in the mammalian brain and play fundamental roles in both brain health and disease [196]. These cells form intricate networks with neural circuits, facilitating the transport of energy metabolites essential for maintaining complex behaviors [197]. Astrocytes possess unique metabolic characteristics that enable metabolite exchange while serving dual roles as energy precursors and paracrine signaling molecules. This phenomenon is termed astrocyte-neuron metabolic coupling [1], which is crucial for regulating synaptic activity, functional plasticity, and behavior [1, 198]. Research advances in the molecular diversity of astrocytes and the coupling properties of astrocyte-neuron metabolism over the years are summarized in Fig. 5.

However, the complexity of astrocyte-neuron coupling presents a major research challenge in elucidating metabolic interactions across different brain regions. Current research in this field faces three key challenges across different dimensions. Spatially, astrocytes exhibit significant metabolic heterogeneity across brain regions, while temporally, metabolic coupling demonstrates dynamic plasticity. From a technical perspective, the field still lacks high spatiotemporal resolution methods for *in* *vivo* metabolic monitoring. To address these challenges, researchers are developing innovative solutions, including the construction of regional metabolic maps and the integration of cutting-edge techniques such as single-cell metabolomics and precision modulation technologies.

In translational medicine, while therapeutic strategies targeting astrocyte-neuron metabolic coupling show considerable promise, most candidate drugs remain in preclinical stages with incomplete safety profiles. Notably, precise optimization of treatment time windows is required to mitigate potential adverse effects. With advancing research and technological progress, these targeted therapies are anticipated to enter clinical trials, offering novel treatment options for neurological disorders. This developmental trajectory underscores the importance of translating basic research into clinical applications while emphasizing the need for cautious and systematic approaches in therapeutic advancement.

Another question worth exploring is whether the metabolic targets discussed here are functionally relevant to specific subtypes of neurons or astrocytes. While many of these targets are broadly expressed across cell types, or specifically expressed within a single cell type, whether they exert subtype-specific effects remains unclear. Emerging evidence from single-cell and spatial transcriptomic studies has revealed unexpected heterogeneity in metabolic gene expression even within classically defined cell types, raising the possibility that certain interventions may exert selective effects on discrete cellular subpopulations [199–202]. Future studies will need to combine single-cell sequencing and spatial transcriptomics to map the expression patterns of these targets across distinct cell subtypes, along with conditional knockout strategies to perturb target function in a subtype-specific manner. Such approaches would help clarify whether the functional effects of these targets are restricted to particular neuronal or astrocytic subtypes.

## Data Availability

No datasets were generated or analysed during the current study.

## Abbreviations

A1R: Adenosine receptor 1
AD: Alzheimer’s disease
ANLS: Astrocyte-neuron lactate shuttle
ApoE: Apolipoprotein E
BLA: Basolateral amygdala
CA: Cornu ammonis
CNS: Central nervous system
Cx30: Connexin 30
Cx43: Connexin 43
DG: Dentate gyrus
EAAT: Excitatory amino acid transporter
FAs: Fatty acids
F2,6BP: Fructose-2,6-bisphosphate
GABA: γ-Aminobutyric acid
GLTs: Glutamate transporters
GLUT: Glucose transport
Gly: Glycine
GS: Glutamine synthetase
HCAR1: Hydrocarboxylic acid receptor 1
HD: Huntington’s disease
HTT: Huntingtin
KBs: Ketone bodies
Lcn2: Lipocalin-2
LDs: Lipid droplets
MCT: Monocarboxylate transporter
NAc: Nucleus accumbens
NMDAR: N-methyl-D-aspartic acid receptor
Nrf2: Nuclear factor erythroid 2-related factor 2
OXPHOS: Oxidative phosphorylation
PD: Parkinson’s disease
PFC: Prefrontal cortex
PKM2: Pyruvate kinase M2
PVN: Paraventricular nucleus
PPP: Pentose phosphate pathway
ROS: Reactive oxygen species
SCZ: Schizophrenia
Ser: Serine
TCA: Tricarboxylic acid
VGLUT: Vesicular glutamate transporter
VMH: Ventromedial hypothalamus
